# Study profile: the Durban Diabetes Study (DDS): a platform for chronic disease research

**DOI:** 10.1017/gheg.2015.3

**Published:** 2016-02-05

**Authors:** T. R. Hird, E. H. Young, F. J. Pirie, J. Riha, T. M. Esterhuizen, B. O'Leary, M. I. McCarthy, M. S. Sandhu, A. A. Motala

**Affiliations:** 1 Department of Medicine, University of Cambridge, Cambridge, UK; 2 Wellcome Trust Sanger Institute, Hinxton, UK; 3 Department of Diabetes and Endocrinology, Nelson R. Mandela School of Medicine, University of KwaZulu-Natal, Durban, South Africa; 4 Faculty of Medicine and Health Sciences, Centre for Evidence-Based Health Care, Stellenbosch University, Stellenbosch, South Africa; 5 Research and Policy Department, Office of Strategy Management, eThekwini Municipality, Durban, South Africa; 6 Oxford Centre for Diabetes, Endocrinology and Metabolism, University of Oxford, Oxford, UK

**Keywords:** Chronic disease, epidemiology, genetics, population-based, study profile

## Abstract

The Durban Diabetes Study (DDS) is a population-based cross-sectional survey of an urban black population in the eThekwini Municipality (city of Durban) in South Africa. The survey combines health, lifestyle and socioeconomic questionnaire data with standardised biophysical measurements, biomarkers for non-communicable and infectious diseases, and genetic data. Data collection for the study is currently underway and the target sample size is 10 000 participants. The DDS has an established infrastructure for survey fieldwork, data collection and management, sample processing and storage, managed data sharing and consent for re-approaching participants, which can be utilised for further research studies. As such, the DDS represents a rich platform for investigating the distribution, interrelation and aetiology of chronic diseases and their risk factors, which is critical for developing health care policies for disease management and prevention. For data access enquiries please contact the African Partnership for Chronic Disease Research (APCDR) at data@apcdr.org or the corresponding author.

## Study rationale

Infectious diseases, nutritional deficiencies and maternal and neonatal conditions are the dominant contributors to the overall burden of disease worldwide [1]. However, the burden of non-communicable diseases (NCDs) such as diabetes, cardiovascular disease and cancer, has increased in the last decade. In 2012, an estimated 2.8 million deaths in sub-Saharan Africa (SSA) were attributable to NCDs; they are projected to be the most common cause of death in SSA by 2030 [2, 3]. The increasing prevalence of diabetes is a key component of the global rise in NCDs. The International Diabetes Federation currently estimates a 54% increase in global prevalence of diabetes, from 382 to 592 million by 2035 [4]. SSA is projected to have a 109% increase in the prevalence of diabetes, from 19.8 million in 2013 to 41.5 million by 2035, representing the highest proportional increase of any region in the world.

The rise in diabetes prevalence is thought to be a consequence of rapid demographic and epidemiological transitions and urbanisation in the region [4, 5]. Within SSA, the prevalence of chronic diseases such as diabetes varies markedly among populations, suggesting that these disorders arise from complex interrelations between environmental and biological factors [6]. Socioeconomic and demographic differences, diagnostic approaches, variation in lifestyle factors and genetic predisposition may explain some of these differences [7–9]. However, there is a lack of large-scale population-based studies that have assessed these systematically in SSA populations. Recent population-based diabetes studies have varied in size and scope (as shown in online Supplementary Table S1); many have only assessed a subset of potential risk factors, limiting comparative analyses. Relatively few have utilised validated survey designs, such as the WHO STEPwise approach to Surveillance (STEPS), to capture a broadened set of variables, as in the DDS. There is, therefore, a need for high-quality data to more reliably assess the burden of chronic diseases and the full spectrum of their risk factors, in order to determine the individual contribution and impact of these risk factors in SSA. In 2013, an estimated 9.3% of the population in South Africa had diabetes, which is one of the highest national prevalences of diabetes in SSA [10]. Studies investigating the prevalence of diabetes in South Africa have reported differing prevalence between urban and rural areas and by ethnicity [11–14]. However, the only study population-based epidemiological study of diabetes in Durban was conducted in the early 1990s and found a prevalence of 5.3% [15].

We therefore established the DDS as a population-based research framework to investigate a broad range of lifestyle, medical and genetic factors and their association with diabetes – the primary research focus of the study – together with other chronic diseases and traits including hypertension, dyslipidaemia, human immunodeficiency virus (HIV) and hepatitis C virus (HCV) in an urban South African (black) population living in the eThekwini Municipality (also known as the city of Durban). The study provides a resource to examine a wide range of research questions in order to inform health policy and practice around the burden and aetiology of chronic diseases; this in turn will enable effective targeting of resources in South Africa and across the region. The DDS also provides a framework for future research including the possibility of interventional studies. The DDS is a collaborative initiative between the University of KwaZulu-Natal, the University of Cambridge, the University of Oxford and the Wellcome Trust Sanger Institute.

## Study population

**Fig. 1.**
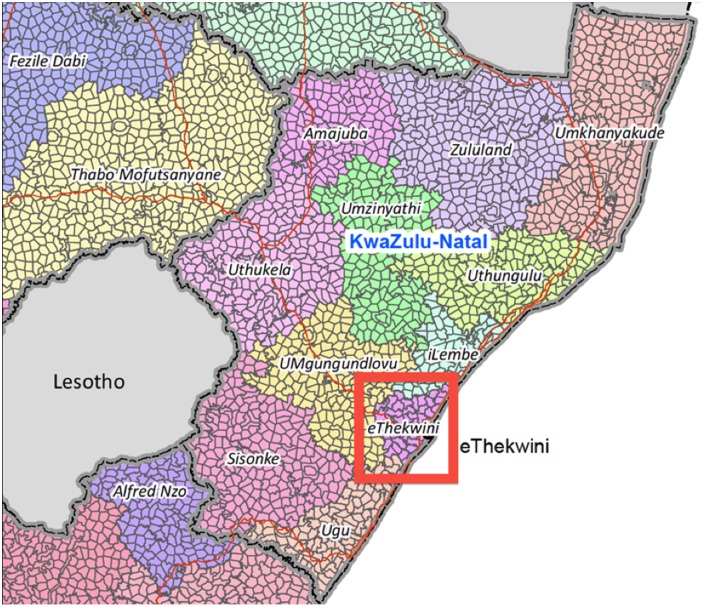
Map of KwaZulu-Natal and eThekwini, South Africa. The map shows the municipal boundaries in KwaZulu-Natal, with the eThekwini Metropolitan Municipality highlighted in red. Adapted from Naudé *et al*. [16].

The study area is the eThekwini Municipality (city of Durban) in KwaZulu-Natal, South Africa as shown in Fig. 1. [16] Based on the 2011 population census for South Africa, black Africans constitute 79.6% (41 000 938) of the total population of 50.8 million [17]. KwaZulu-Natal is the second most densely populated province in South Africa, accounting for around one-fifth of the total population (10 267 300), of whom 86.8% are black African, 52.3% are female and 73.7% are aged 35 years or older [18].

The DDS sampling frame was developed independently by the Research and Policy Department of the eThekwini Municipality, and is divided into nine planning unit clusters (PUCs), representing nine townships (Umlazi, Inanda, KwaMashu, Ntuzuma, Mpumalanga Complex, Cato Manor, Clermont, Lamontville and Chesterville). Geographic information system mapping techniques were used to gather accurate geographical and boundary data on the selected PUC areas. The study region is predominantly urban with a total population of approximately 1 378 750 individuals, 61% of whom live in informal dwellings. In each PUC streets are randomly selected, with the number of streets selected being proportional to the size of the PUC population. Next, within each randomly selected street, households are nominated by systematic cluster sampling, with the number of formal or informal houses selected within each street proportional to the ratio of formal to informal housing in that PUC. Further, in each street the number of selected houses is oversampled, allowing for up to 20% non-participation without compromising overall study size. Within each nominated household, all family members who are of African descent, not pregnant and aged 18 years or older are eligible for recruitment into the study.

Before participant recruitment and data collection begin, PUC areas are sensitised to the study using flyers, neighbourhood-specific community activation events and reminders for local residents. These stages of community engagement and mobilisation are conducted with the support of the provincial department of health (KwaZulu-Natal), local health authorities and community leaders. Once the study rationale and procedures have been explained, all eligible individuals present at the time of mobilisation are invited to participate in the study. A non-responder is defined as an individual who has declined an invitation to participate; a non-response household is defined as a household who has been contacted on at least three occasions with all habitants absent on each occasion. Each non-response household is replaced by another house pre-selected from the same street as described above. Data and sample collection are undertaken at pre-arranged study appointments at a central community venue. All study appointments occur in the morning; this is essential as participants are required to fast overnight for a 75 g oral glucose tolerance test (OGTT).

## Statistical power

The DDS sample size – based on population size, prevalence precision estimates and power for epidemiological and genetic association studies – is 10 000 participants. Previous studies in SSA have estimated a diabetes prevalence of around 10% (which is consistent with preliminary findings from our study; data not shown). Based on this estimate, and taking into account design effects, our sample size is sufficient to estimate the population prevalence of diabetes with a 95% confidence interval (CI) of less than 1%. For logistic regression analyses that involve diabetes as an outcome, based on 1000 cases and 9000 controls, the study has more than 80% power to detect odds ratios (ORs) as low as 1.2 for exposure prevalences equal to 30%. For exposure prevalences greater than 30%, ORs less than 1.2 can be detected with the same power. Further, for exposure prevalences as low as 5% the study will have more than 80% power to detect ORs of at least 1.5. At this sample size we will also have the ability to examine genetic variants that explain 0.5 % of the variation in a relevant trait with more than 80% power using a genome-wide statistical threshold.

## Data collection

The DDS collects detailed questionnaire information on participant health, lifestyle and socioeconomic indices, as well as biophysical measurements, biochemical, haematological and serologic biomarkers for non-communicable and infectious diseases, and genetic information, as shown in Table 1. The questionnaire used in this study is an adaptation of the standardised and validated WHO STEPS tool designed for the collection of disease risk factors [19]. The questionnaire responses are collected on an electronic data capture system [electronic questionnaire (EQ)]. For increased accuracy and efficiency the EQ has built-in data checks, including double entry of numbers, plausibility of answers and appropriate numerical limitations, the detailed methods of which have been previously described [20]. Biophysical measurements (height, weight, waist and hip circumferences and blood pressure) and samples of venous blood (baseline fasting sample, 30- and 120-min) and urine are collected in the same study appointment to maximise efficiency. Adhering to standardised protocols, trained study personnel perform all study procedures and sample collections. Study equipment is calibrated regularly. Blood and urine samples are stored in cold boxes maintained at 4–8 °C until transported to a laboratory within 6 h of collection. Full blood count, plasma glucose and HbA_1c_ analyses are undertaken on the day of sample collection, at a local private laboratory. All other samples are transported to the University of KwaZulu-Natal laboratory and processed for storage (including centrifugation, separation into serum and plasma, and DNA extraction). The processed samples are stored at −70 °C for future analyses. Extracted DNA is sent to the Wellcome Trust Sanger Institute in the UK for next-generation genomic analyses.

**Table 1.** Planned data component domains in the DDS

Domain	Components
Health and lifestyle questionnaire	Demographic information
	Education, occupation and livelihood
	Socioeconomic indices (household features and ownership)
	Tobacco use
	Alcohol consumption
	Dietary behaviour
	Physical activity
	Medical history
	Family medical history
Biophysical measurements	Blood pressure and heart rate
	Height and weight
	Waist circumference and hip circumference
NCD & infectious disease biomarkers and genetic data	
Glycaemic biomarkers	OGTT:
	0-, 30-, 120-min plasma glucose
	0-, 30-, 120-min serum insulin
	HbA_1c_
Cardiometabolic and haematological biomarkers	Full blood count
	Total cholesterol
	LDL
	HDL
	Lp(a)
	Triglycerides
	Aspartate amino transferase
	Alanine amino transferase
	Alkaline phosphatase
	Gamma glutamyl transferase
	Bilirubin
	Albumin
	Albumin–creatinine ratio (urine sample)
	Sodium and potassium (urine sample)
Infectious disease biomarkers	HIV
	HBV
	HCV
Genetic markers (host and pathogen)	Illumina SNP chip arrays
	Illumina whole genome and exome sequencing
	Viral whole genome sequencing

NCD, non-communicable disease; OGTT, oral glucose tolerance test; HbA_1c_, glycated haemoglobin; LDL, low-density lipoprotein; HDL, high-density lipoprotein; Lp(a), lipoprotein(a); HIV, human immunodeficiency virus; HBV, hepatitis B virus; HCV, hepatitis C virus; SNP, single-nucleotide polymorphism.

Feedback of results to the participants starts during the study appointment. All participants receive blood pressure and body mass index results immediately, accompanied by appropriate lifestyle and health guidance from trained study personnel including advice to seek medical referral. Results from the OGTT are posted to participants, unless they choose not to receive them. Participants may also choose to receive the results of other tests such as HIV, HCV and liver function tests, by appointment at the DDS feedback office. Where participant results are abnormal, according to clinical algorithms defined in accordance with national guidelines, participants are advised to seek further assessment (investigation and management) at the appropriate local health facility as part of their standard clinical care.

## Ethical considerations

The DDS has obtained full ethical approval from the Biomedical Research Ethics Committee at the University of KwaZulu-Natal (reference: BF030/12) and the UK National Research Ethics Service (reference: 14/WM/1061). Written informed consent is obtained from all study participants. The consent form covers the following activities: data collection by questionnaire and biophysical measures, sample collection and analysis including linked-anonymous HIV testing, feedback of results, consent for DNA extraction and genomic analyses, consent for use of data and samples for future research, and permission to re-approach participants for new studies.

## Study progress

**Fig. 2.**
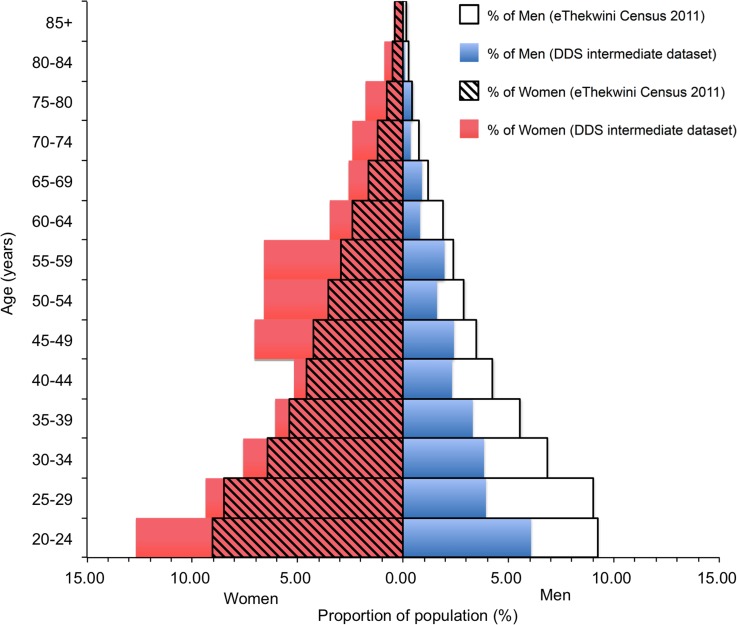
Population pyramid comparing the age and sex structure of the DDS intermediate dataset (*n* = 1204) with the 2011 census population for the eThekwini municipality (city of Durban). DDS data for women (red bars) and men (blue bars) are superimposed over census data for women (black and white bars, hatched) and men (black and white bars, plain).

**Fig. 3.**
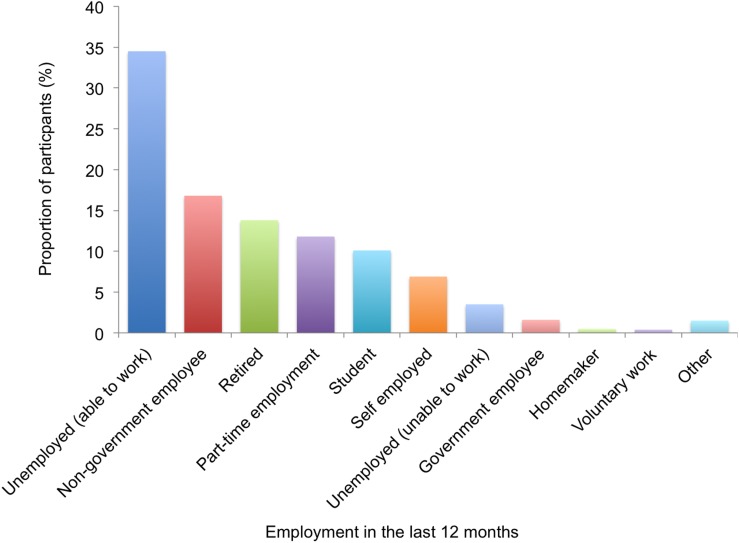
Proportion of participants in the DDS intermediate dataset (*n* = 1204) by category of employment in the preceding 12 months.

Data collection began in November 2013. Through successful community sensitisation, engagement and mobilisation, the study has achieved a high level of participation: to date, 1300 individuals have been invited join the study, of whom 96 refused participation, with a resulting response of 92.6% (*n* = 1204) (Table 2). The age and sex structure of this DDS intermediate dataset are compared with the 2011 census data for the eThekwini population in Fig. 2 [18]. Of the 1204 participants recruited to date, 28.3% (341) are men compared with 48.2% found in the general population census data. This low proportion of men in the DDS is consistently observed in population-based studies in South Africa. Likely explanations include high levels of unemployment in the townships sampled leading to men moving away for work (migrant labour system) [21, 22], and challenges in participant mobilisation as male potential participants are often away during office hours when the study mobilisation takes place [11–14]. The age distribution of the DDS intermediate dataset is broadly similar to the general population of eThekwini: 46.6% were aged 18–34 years in the DDS compared with 49.1% in the 2011 eThekwini census; 44.1% (DDS) compared with 43.6% (census) were aged 35–64 years; and 9.2% (DDS) compared with 7.3% (census) were aged 65 years or older. The employment status of DDS participants for the preceding 12 months is shown in Fig. 3: 37.2% were in full- or part-time employment, 13.9% were retired, and 10.2% were students. Thirty-eight per cent of the DDS population reported being unemployed (including both individuals able and unable to work), compared with 30.2% in the 2011 eThekwini census.

**Table 2.** Demographic characteristics of participants in the DDS intermediate dataset (n = 1204)

Variable	*N* (%)
Sex	
Men	341 (28.3)
Women	863 (71.7)
Age category (years)	
18–24	284 (23.6)
25–34	277 (23.0)
35–44	189 (15.7)
45–54	198 (16.4)
55–64	144 (12.0)
65+	111 (9.2)
Do not know	1 (0.1)
Language	
isiZulu	1084 (90.0)
isiXhosa	79 (6.6)
Sesotho	28 (2.3)
Other	13 (1.1)
Level of education	
Nil	16 (1.3)
Nursery or preschool	17 (1.4)
Primary	246 (20.4)
Secondary	848 (70.5)
Post-secondary/vocational	19 (1.6)
University	52 (4.3)
Do not know	6 (0.5)

## Strengths and limitations

The DDS is a platform for population-based research encompassing the distribution, interrelation and aetiology of chronic diseases and their risk factors in an urban South African setting, providing a valuable research resource and creating an evidence base to inform local and national health policy and public health programmes. The study is designed to characterise a wide range of phenotypes – including banked biological samples for future analyses – and to align these with genomic data. In this way, the DDS has the scope to address a broad set of research questions and the ability to provide aetiological insights into the variation in risk factors for diabetes and other chronic diseases among adults in this setting. The study has no upper age limit for participants and is therefore well placed to investigate disease and risk factors in older populations [23]. The support of community leaders and local residents, cultivated through a careful and involved dialogue with these communities, enables the recruitment of informed and confident participants who are willing to enter a survey containing sensitive questions and encourages a high proportion of responders.

A limitation of the DDS is its cross-sectional design; it is therefore not possible to assess incidence in this study or the temporal association between exposures and outcomes. However, the DDS has obtained consent to follow up and re-approach participants, thus enabling the potential for longitudinal studies, prospective case ascertainment of incident disease, and interventional studies. The data collection framework of the DDS has been standardised and aligned to the data collection framework of both the APCDR and the pan-African Human Heredity and Health in Africa (H3A) diabetes study [24]. This will allow future comparison with other SSA countries, including pooled analyses of equivalent data from existing studies (as shown in online Supplementary Table S1) to better characterise the epidemiology of diabetes in SSA.

The study specifically recruits participants of African descent in order to better assess the association between exposures and disease in this population [25, 26]. By focusing on a single ethnic group, the DDS may limit its generalisability to other populations; this highlights the importance of conducting similar studies nationally and across SSA to fully understand the aetiology and determinants of chronic disease. However, the intermediate dataset presented here indicates that the DDS population is broadly representative of its target population.

## Future plans

The DDS continues to recruit participants towards the sample size of 10 000 participants, with sufficient statistical resolution to present accurate prevalence estimates and identify and assess epidemiological associations between risk factors and disease that are informative for policy-makers and public health programmes in the region. Given the planned sample size, the DDS will be powered to detect less prevalent exposures, and distinguish more subtle associations between risk factors and disease. The final sample size will also have power to identify subpopulations at high risk of chronic diseases, whilst acknowledging all the caveats of undertaking such subgroup analyses. Additionally, with the option to re-approach participants, the DDS has the potential as a platform for future research including studies of diabetes complications and intervention studies.

A central aim of the DDS is to combine epidemiological methods with population-based genome-wide technologies. This integration has already provided new insights into the biology of chronic diseases and their risk factors in Western populations [27]. However, the relevance of many recent genomic findings to populations in SSA is not known. Given the marked genomic diversity among populations in SSA, understanding the genomic basis for chronic diseases and their risk factors in populations of African descent is likely to provide additional insights into the genetic determinants of chronic diseases and potential therapeutic strategies [8]. The DDS is thus well positioned to take advantage of these opportunities to conduct genomic analyses and contribute to the global genomics research arena.

## Data access

All data (health, lifestyle and socioeconomic questionnaire, biophysical measurements, NCD and infectious disease biomarkers and genetic) generated in the DDS are stored and curated as part of the APCDR. For data access inquiries you may contact the APCDR data access committee (data@apcdr.org).

## Supplementary material

For supplementary material accompanying this paper visit http://dx.doi.org/10.1017/gheg.2015.3 click here to view supplementary material
