# Supplementary material for: Study profile: the Durban Diabetes Study (DDS): a platform for chronic disease research
Source: Glob Health Epidemiol Genom. 2016 Feb 5;1:e2. doi: 10.1017/gheg.2015.3 (PMC5732575; doi:10.1017/gheg.2015.3)
Supplement: Supplementary file 1 [file S2054420015000032sup001.docx]

**Supplementary Table 1:** Selected population-based prevalence studies on diabetes in black populations in sub-Saharan Africa, 2000-2015

| **Country** | **Author, year** | **Rural/Urban** | **Sample size** | **Age (years)** | **Method** | **Age-adjusted Prevalence (%)**  **(95% CI)** | | | **Data collected** |
| --- | --- | --- | --- | --- | --- | --- | --- | --- | --- |
|  |  |  |  |  |  | **All** | **Men** | **Women** |  |
| **Tanzania** | Aspray (2000) (1) | Urban  Rural | 770  928 | ≥15 | FCBG | -  - | 5.9  1.7 | 5.7  1.1 | Demographic information, Anthropometric measurements, physical activity |
| **Cameroon** | Sobngwi (2002) (2) | Urban  Rural | 1183  1282 | ≥15 | FCBG | -  - | 6.2  (3.7-8.9)  4.7  (2.5-6.9) | 4.7  (2.6-6.8)  2.9  (1.5-4.4) | Demographic information, Anthropometric measurements, blood pressure, physical activity |
|  | MOH CAMBOD (2004) (3) | Urban | 10,824 | ≥15 | OGTT | 6.06 | 6.4 | 5.7 | (WHO STEPS based) Demographic information, occupation, education, tobacco consumption, alcohol consumption, nutrition, physical activity, anthropometric measurements, blood pressure |
|  | Katte (2014) (4) | Urban | 1702 | ≥18 | FCBG | - | 3.3  (1.5-5.1) | 5.6  (3.5-7.7) | (WHO STEPS based) Demographic information, education, tobacco consumption, alcohol consumption, physical activity, anthropometric measurements, blood pressure |
| **Ghana** | Amoah (2002) (5) | Urban | 4733 | ≥25 | FCBG | 6.4 | 7.7 | 5.5 | Demographic information, anthropometric measurements, blood pressure |
| **Nigeria** | Nyenwe (2003) (6) | Urban | 502 | ≥40 | OGTT | 7.9 | 9.1 | 6.3 | Demographic information, family history of diabetes, tobacco consumption, alcohol consumption, nutrition, physical activity, anthropometric measurements, blood pressure |
|  | Oladapo (2010) (7) | Rural | 2000 | 18-64 | FCBG | 2.5 | 2.1 | 2.8 | (WHO STEPS based) Demographic information, education, occupation, tobacco consumption, alcohol consumption, nutrition, physical activity, anthropometric measurements, blood pressure, cardiometabolic biomarkers (lipids) |
|  | Ejim (2011) (8) | Rural | 858 | 40-70 | FCBG | 4.4 | 7.3 | 3.3 | Demographic information, anthropometric measurements, blood pressure, cardiometabolic biomarkers (lipids) |
| **South Africa** | Alberts (2005) (9) | Rural | 2106 | ≥30 | FCBG | - | 8.5 | 8.8 | Demographic information, education, occupation, tobacco consumption, alcohol consumption, nutrition, physical activity, anthropometric measurements, blood pressure, cardiometabolic biomarkers (lipids, liver function tests) |
|  | Motala (2008) (10) | Rural | 1025 | ≥15 | OGTT | 3.9 | 3.5 | 3.9 | Demographic information, family history of diabetes, tobacco consumption, alcohol consumption, physical activity, anthropometric measurements, blood pressure, cardiometabolic biomarkers (lipids) |
|  | Peer (2012) (11) | Urban | 1099 | 25-74 | OGTT | 13.1 | 11.3 | 14.7 | (WHO STEPS based) Demographic information, education, occupation, tobacco consumption, alcohol consumption, nutrition, physical activity, anthropometric measurements, blood pressure, cardiometabolic biomarkers (lipids) |
| **Zimbabwe** | MOH STEPS (2005) (12) | Pop Rep | 3081 | ≥25 | FCBG | 10.0 | - | - | (WHO STEPS) Demographic information, education, occupation, tobacco consumption, alcohol consumption, nutrition, physical activity, anthropometric measurements, blood pressure, cardiometabolic biomarkers (lipids) |
| **Guinea** | Balde (2007) (13) | Urban  Rural | 886  651 | ≥35 | FCBG | 6.7  (5.1-8.3)  5.3  (3.6-7.0) | - | - | Demographic information, family history of diabetes, anthropometric measurements, blood pressure |
|  | Camara (2015) (14) | Pop Rep | 2491 | ≥15 | FCBG | 3.3  (3.2-3.3) | 3.7  (3.7-3.8) | 3.5  (3.4-3.5) | (WHO STEPS) Demographic information, education, occupation, tobacco consumption, alcohol consumption, nutrition, physical activity, anthropometric measurements, blood pressure, cardiometabolic biomarkers (lipids) |
| **Kenya** | Chistensen (2009) (15) | Urban  Rural | 281  1178 | ≥17 | OGTT | 12.2  (5.4-23.2)  2.2  (0.8-5.2) | -  - | -  - | Demographic information, family history of diabetes, tobacco consumption, alcohol consumption, nutrition, physical activity, anthropometric measurements, blood pressure, cardiometabolic biomarkers (lipids), ultrasonography (fat thickness) |
| **Zambia** | Nsakashalo-Senkwe (2011) (16) | Urban | 1928 | ≥25 | FCBG | 2.7 | - | - | (WHO STEPS based) Demographic information, education, occupation, tobacco consumption, alcohol consumption, nutrition, physical activity, anthropometric measurements, blood pressure, cardiometabolic biomarkers (lipids) |
| **Senegal** | Duboz (2012) (17) | Urban | 600 | ≥20 | FCBG | 17.9 | 14.0 | 21.8 | Demographic information, education, anthropometric measurements, blood pressure |
|  | Pessinaba (2013) (18) | Urban | 1424 | ≥15 | FCBG | 10.4  (8.9–12.1) | - | - | (WHO STEPS based) Demographic information, education, occupation, tobacco consumption, alcohol consumption, nutrition, physical activity, anthropometric measurements, blood pressure, cardiometabolic biomarkers (lipids) |
| **Togo** | Baragou (2012) (19) | Urban | 2000 | ≥18 | FCBG | 7.3 | 6.9 | 7.3 | (WHO STEPS based) Demographic information, education, occupation, tobacco consumption, alcohol consumption, nutrition, physical activity, anthropometric measurements, blood pressure, cardiometabolic biomarkers (lipids) |
| **Uganda** | Mayega (2013) (20) | Rural | 1497 | 35-60 | FCBG | 7.4 | 6.5 | 8.1 | (WHO STEPS based) Demographic information, family history of diabetes, education, occupation, tobacco consumption, alcohol consumption, nutrition, physical activity, anthropometric measurements, blood pressure |
| **Malawi** | Msyamboza  (2014) (21) | Pop Rep | 3056 | 25-64 | FCBG | 5.6  (2.6-8.5) | 6.5  (2.6-10.3) | 4.7  (2.4-7.0) | (WHO STEPS based) Demographic information, education, occupation, tobacco consumption, alcohol consumption, nutrition, physical activity, anthropometric measurements, blood pressure, cardiometabolic biomarkers (lipids) |

This table describes 21 population-based diabetes prevalence studies (using the current WHO(1998) diagnostic criteria) from 13 SSA countries that have been published in the past 15 years. Prevalence estimates are presented, where possible, with 95% confidence intervals. **Abbreviations:** 95% CI, 95% confidence intervals; FCBG, Fasting Capillary Blood Glucose; OGTT, Oral Glucose Tolerance Test; Pop Rep, sample representative of the national population.

**References**

1. **Aspray T. J., et al.** Rural and urban differences in diabetes prevalence in Tanzania: the role of obesity, physical inactivity and urban living. Trans R Soc Trop Med Hyg. 2000;94(6):637-44.

2. **Sobngwi E., et al.** Physical activity and its relationship with obesity, hypertension and diabetes in urban and rural Cameroon. Int J Obes Relat Metab Disord. 2002;26(7):1009-16.

3. **MOH Cameroon**. Cameroon Burden of Diabetes Project (Cambod): Baseline Survey Report. Ministry of Health, Cameroon, 2004.

4. **Katte J. C., et al.** Coincidence of diabetes mellitus and hypertension in a semi-urban Cameroonian population: a cross-sectional study. BMC Public Health. 2014;14:696.

5. **Amoah A. G., Owusu S. K. and Adjei S.** Diabetes in Ghana: a community based prevalence study in Greater Accra. Diabetes Res Clin Pract. 2002;56(3):197-205.

6. **Nyenwe E. A., et al.** Type 2 diabetes in adult Nigerians: a study of its prevalence and risk factors in Port Harcourt, Nigeria. Diabetes Res Clin Pract. 2003;62(3):177-85.

7. **Oladapo O. O., et al.** A prevalence of cardiometabolic risk factors among a rural Yoruba south-western Nigerian population: a population-based survey. Cardiovasc J Afr. 2010;21(1):26-31.

8. **Ejim E. C., et al.** Prevalence of cardiovascular risk factors in the middle-aged and elderly population of a nigerian rural community. J Trop Med. 2011;2011:308687.

9. **Alberts M., et al.** Prevalence of cardiovascular diseases and associated risk factors in a rural black population of South Africa. Eur J Cardiovasc Prev Rehabil. 2005;12(4):347-54.

10. **Motala A. A., et al.** Diabetes and other disorders of glycemia in a rural South African community: prevalence and associated risk factors. Diabetes Care. 2008;31(9):1783-8.

11. **Peer N., et al.** Rising diabetes prevalence among urban-dwelling black South Africans. PLoS One. 2012;7(9):e43336.

12. **Ministry of Health (MOH) Zimbabwe**. National Survey: Zimbabwe Non-Communicable Disease Risk Factors (ZiNCoDs). Preliminary Report. : Ministry of Health and Child Welfare, Zimbabwe, 2005.

13. **Balde N. M., et al.** Diabetes and impaired fasting glucose in rural and urban populations in Futa Jallon (Guinea): prevalence and associated risk factors. Diabetes Metab. 2007;33(2):114-20.

14. **Camara A., et al.** High prevalence, low awareness, treatment and control rates of hypertension in Guinea: results from a population-based STEPS survey. J Hum Hypertens. 2015.

15. **Christensen D. L., et al.** Prevalence of glucose intolerance and associated risk factors in rural and urban populations of different ethnic groups in Kenya. Diabetes Res Clin Pract. 2009;84(3):303-10.

16. **Nsakashalo-Senkwe M., et al.** Combined prevalence of impaired glucose level or diabetes and its correlates in Lusaka urban district, Zambia: a population based survey. Int Arch Med. 2011;4(1):2.

17. **Duboz P., et al.** Prevalence of diabetes and associated risk factors in a Senegalese urban (Dakar) population. Diabetes Metab. 2012;38(4):332-6.

18. **Pessinaba S., et al.** Prevalence and determinants of hypertension and associated cardiovascular risk factors: data from a population-based, cross-sectional survey in Saint Louis, Senegal. Cardiovasc J Afr. 2013;24(5):180-3.

19. **Baragou S., et al.** Prevalence of cardiovascular risk factors in an urban area of Togo: a WHO STEPS-wise approach in Lome, Togo. Cardiovasc J Afr. 2012;23(6):309-12.

20. **Mayega R. W., et al.** Diabetes and pre-diabetes among persons aged 35 to 60 years in eastern Uganda: prevalence and associated factors. PLoS One. 2013;8(8):e72554.

21. **Msyamboza K. P., Mvula C. J. and Kathyola D.** Prevalence and correlates of diabetes mellitus in Malawi: population-based national NCD STEPS survey. BMC Endocr Disord. 2014;14:41.
